# Commentary on "The relationship between risky sexual behaviors and sexual health literacy and self-esteem in young women"

**DOI:** 10.1590/1806-9282.20250646

**Published:** 2025-09-19

**Authors:** Buğra Çetin

**Affiliations:** 1 Altınbaş University, Medical Park Bahçelievler Hospital, Department of Urology – Istanbul, Turkey.

Dear Editor,

I read with great interest the article titled "The relationship between risky sexual behaviors and sexual health literacy and self-esteem in young women" by Ozdemir and Cevik. This research addresses a highly relevant and sensitive topic, offering valuable insights into young women's sexual health, especially within the sociocultural landscape of Turkey. Such contributions are critical for shaping effective public health strategies in societies undergoing cultural transformation.

That said, I would like to respectfully share a few comments that may help further contextualize and enrich the interpretation of the findings. First, the sample in this study was limited to participants with internet access and the ability to engage in online surveys. This may inadvertently exclude certain socioeconomic or cultural groups, potentially affecting the representativeness and generalizability of the results. A more inclusive sampling strategy could enhance the external validity of future research.

Additionally, the use of standardized instruments—particularly the Premarital Sexual Behavior Assessment Scale for Young Women (PSAS-YW)—should be carefully interpreted within the cultural context. In societies such as Turkey, where sexuality is shaped by strong social, religious, and moral norms, individuals may respond to self-report questionnaires in ways influenced by social desirability bias^1^. While the Turkish version of the scale has undergone a validity and reliability study, the absence of a defined cut-off score leaves room for ambiguity in interpreting the levels of "risky" behavior^2^. For culturally sensitive subjects such as sexuality, the inclusion of qualitative components—such as open-ended questions or focus groups—can meaningfully complement quantitative findings by allowing participants to express nuanced perspectives^3^.

Furthermore, the study's finding of no significant relationship between self-esteem and risky sexual behaviors warrants deeper consideration. While this outcome may appear inconsistent with previous literature suggesting a negative association between self-esteem and risky behavior^4^, it might be explained by the age characteristics of the sample. Since most participants were in early adulthood, they may be beyond the more vulnerable stages of adolescent identity formation, where self-esteem typically exerts a stronger influence^5^. Additionally, self-esteem may affect sexual behavior not directly, but rather through mediating variables such as peer pressure, substance use, or coping strategies^6^. Future studies might benefit from exploring such indirect effects through structural equation modeling or other advanced analytical frameworks^7^.

In conclusion, I commend the authors for their valuable contribution to this important field. The findings offer a useful foundation for future studies and public health initiatives. I hope that upcoming research will continue to explore broader psychosocial and cultural dimensions to further deepen our understanding of sexual health behaviors in young women.

## Data Availability

The datasets generated and/or analyzed during the current study are available from the corresponding author upon reasonable request.
